# Exposure of Pharmacists and Pharmacy Technicians to Violence in Community Pharmacies in Southeast Europe: Frequency and Ethical Considerations

**DOI:** 10.3390/pharmacy12030088

**Published:** 2024-06-04

**Authors:** Monika Popčević, Tanja Javorina, Miljenko Košiček, Arijana Meštrović

**Affiliations:** 1 ZU Ljekarna Marušić, 10000 Zagreb, Croatia; monika@ljekarna-marusic.hr; 2 Independent Researcher, 10000 Zagreb, Croatia; tanja.javorina@gmail.com; 3 Independent Researcher, 10000 Zagreb, Croatia; 4 Pharma Expert Consultancy and Education, 10000 Zagreb, Croatia

**Keywords:** workplace violence, community pharmacy, work safety, pharmacists’ attrition, robbery, ethics in healthcare

## Abstract

Pharmacists and pharmacy technicians working in community pharmacies are exposed to the risk of violence in their workplaces. Studies have shown that workplace violence is affecting their job satisfaction, productivity, and mental health. This study aims to identify the frequency of different types of violence, as well as the common perpetrators that community pharmacy staff in SEE (Southeast Europe) are dealing with. A cross-sectional study was conducted using an online questionnaire created for this purpose. Selected community pharmacies in Croatia, Serbia, Bosnia and Herzegovina, and Montenegro participated in this study. In total, 732 responses were collected from 24 pharmacy chains or independent pharmacies including all community pharmacy staff. More than 80% of pharmacists and pharmacy technicians reported having been exposed to verbal violence at the workplace, while more than 20% of them reported physical and sexual violence in the preceding 12 months. There were no statistically significant differences between pharmacists and pharmacy technicians, gender, age groups, or countries in relation to exposure to physical, verbal, and sexual violence. The most common perpetrators were identified as patients/clients. More than 90% of pharmacy staff reported they did not receive any kind of support from their employer nor any other help after experiencing a robbery. There is a need for a structured approach to addressing violence in pharmacies including organized support for pharmacy staff. Achieving quality patient care, despite dealing with violent individuals or situations daily, is one of the greatest ethical challenges for healthcare providers in community pharmacies to be empowered.

## 1. Introduction

Healthcare has been globally recognized as the sector with very high rates of workplace violence (WPV) [1,2]. The European Commission defines workplace violence as “incidents where staff are abused, threatened or assaulted in circumstances related to their work, including commuting to and from work, involving an explicit or implicit challenge to their safety, well-being or health” [3]. Previous studies have shown the impact of violent incidents on stress, decreased job satisfaction, motivation, burnout, sleep, and mental health problems in healthcare [4,5,6,7,8,9,10]. In addition to healthcare workers, WPV affects their employers and organizations, as it might decrease job commitment and productivity. WPV was also recognized as one of the factors that influences the retention of healthcare workers [6,10]. Even more importantly, it affects the quality of health care delivered to patients [2].

There is concerning evidence in the literature that the incidence of violence against healthcare workers is increasing worldwide [10]. Studies and reviews have mainly described WPV among physicians and nurses working in hospitals, while data concerning violence toward pharmacists and pharmacy technicians in community pharmacies is less available.

Community pharmacies are an important part of any healthcare system. Pharmacists are recognized as the most accessible healthcare professionals in primary care settings [11]. They are trusted providers of many important services: dispensing prescription and over-the-counter medicines, counseling patients on the rational use of medicines, compounding, etc. Recently, there has been an increasing trend to embrace the additional roles and services pharmacists are providing, such as vaccination, conducting point-of-care tests, establishing collaborative practices, and organizing public health campaigns [12,13]. Their contribution to society and public health was essential in emergency situations such as the COVID-19 pandemic. In challenging conditions, pharmacies managed to ensure continuous medicine supply. Additionally, they have established new practices in adaptation to the new community needs, such as telepharmacy counseling, home delivery services, providing education and evidence-based advice for patients regarding COVID-19, etc. [14].

The pharmacy work setting is unique and differs from other healthcare sectors, but the significant occupational risk related to the high rates of exposure to workplace violence is similar to others. Community pharmacies have open access to the public. No prior arranged appointments are required, and no waiting rooms are available to reduce the possibility of violent behavior by the visitors. In one systematic review, it was estimated that half of the pharmacists working in the community pharmacies faced WPV, which is more than healthcare workers collectively [15]. In a study among pharmacists in Ireland [16], 77% of respondents experienced some violent incident in the preceding 12 months. A systematic review of WPV toward pharmacists showed that verbal violence is the most frequent type of violence in the community pharmacy [15]. Other forms observed were sexual as well as physical violence. According to existing research from Europe, Australia, Africa, and UAE, patients/clients were recognized as common perpetrators of violence [16,17,18,19,20,21].

The underreporting of violent incidents in the whole healthcare sector is described in the literature. There is also a lack of well-established organizational support for healthcare workers when they experience violence [10,16,22]. Many healthcare professionals take the incidents of violence as a regular part of their everyday job [19,22]. One study among nurses in five European countries showed that more than 60% of participants (*n* = 1089) stated that reporting violence would be useless [23]. A study from Australia showed that more than half of all respondents (*n* = 248) did not report their experience of violence, except for robberies [16]. Reporting or communication about violent incidents to work colleagues, friends, and family was registered, but reports were not always made to supervisors or the police [16,19,23].

Robbery as an occupational risk is specific to the community pharmacy setting, as it is unlikely to happen in other healthcare sectors. A study from the United Kingdom showed that robberies were frequent in pharmacies [17]. Peterson in Australia [16] stated that pharmacists rarely received any kind of support after violent incidents, including robberies. Feeling safe at the workplace is crucial for quality health care. Experiencing WPV has been negatively correlated with feeling safe at work among nurses and physicians [24,25]. Studies also found that various educational activities, such as training in de-escalation of violent situations, training in communication skills, and implemented protocols and support, were helpful in ensuring safety at workplaces [26,27].

Differences in WPV between genders have been investigated, but results were inconsistent [7,14]. In addition to external violence—where perpetrators were clients, patients, and other third parties—the literature referred to internal types of violence where the perpetrators were working colleagues and supervisors [27].

There are several studies available on violence in pharmacies, which were conducted in different countries before the COVID-19 pandemic. For the Southeast Europe region, there is, to date, no published data on external or internal WPV directed at pharmacy staff. This is the first cross-sectional study investigating the frequency of physical, verbal, and sexual violence directed at pharmacists and pharmacy technicians in the region. This region has a special political and cultural background. The countries are still in transition to a fully democratic system, especially concerning the shift from public to private ownership of pharmacies. There is little attention paid to organizational culture, human resources, and risk management. In addition, in the SEE region, the healthcare systems are overburdened. There is a shortage of general practitioners, and access is limited. These situations often lead to frustration among patients. In most of the countries in the region, there is no required minimum distance between pharmacies, and there is unlimited number of pharmacies in relation to the population. It is difficult for employers to retain staff as there is a lot of competition. Leadership skills are not included in the pharmacy curricula. The roles of team members in community pharmacy are often not clearly defined. In addition, there is no active pharmacists’ union in the countries of the region. There is a lack of professional negotiators committed to improving workers’ rights, workplace safety, and equal pay. The SEE region also has very specific cultural, religious, ethnic, and social diversity in the community, as well as challenging communication issues after the war events in the late nineties.

The study aims to discuss ethical considerations concerning WPV, including reporting of violent events and the support that pharmacists and pharmacy technicians receive from their organizations. For this purpose, the following hypotheses were put forward:

1. The most common type of workplace violence in pharmacies in Southeast Europe is verbal violence.
2. The most common perpetrators of WPV in pharmacies in Southeast Europe are patients/clients.
3. Female workers are more frequently exposed to WPV than male workers.
4. In the majority of cases, pharmacists and pharmacy technicians talk about experiencing physical violence, and in the minority of cases, they talk about experiencing sexual violence.
5. Pharmacists and pharmacy technicians talk more frequently to their work colleagues about the violence they have experienced compared to the other interlocutors.
6. Most pharmacists and pharmacy technicians who have experienced a robbery at their workplace consider it to be a very traumatic experience.
7. Most pharmacists and pharmacy technicians who have experienced a robbery at their workplace receive no support from their employer afterward nor any other kind of organizational support.
8. Pharmacists and pharmacy technicians who are more frequently exposed to WPV feel less safe at work.
9. Exposure to WPV among pharmacists and pharmacy technicians is connected to considering a job change.

For each type of violence, the following definitions were used:

Physical violence means the use of physical force against another person or group of people that results in physical, sexual, or psychological harm. This includes hitting, slapping, stabbing, shooting, pushing, pinching, throwing objects, etc. [2].

Verbal violence means a form of psychological violence that includes threats, insults, belittling, shouting, intimidation, humiliation, and the use of abusive speech or profanities.

Sexual violence means any sexual act, attempted sexual act, unwanted sexual comment, or suggestion directed against a person and their sexuality that can be committed by another person regardless of their relationship to the victim or their situation. This also includes sexual harassment: unwanted verbal innuendos, unwanted phone calls and touching, undue attention, standing too close, staring, emotional stalking, etc. [2].

## 2. Materials and Methods

For the purpose of this cross-sectional study, a questionnaire was created. Questions included were partly based on other available validated, free-for-use questionnaires constructed for research on workplace violence against healthcare workers [28]. Since the pharmacy setting is different from other healthcare settings, the majority of questions were, however, designed for this study specifically. It was peer reviewed by a group of ten experts in pharmacy from all countries included in the study. A psychologist was also part of the group in order to cover all important aspects of the topic as well as to form comprehensible and unambiguous questions. The final version of the questionnaire consisted of 30 questions divided into four groups (Supplementary Materials, File S1). It was designed to collect data on three types of workplace violence, determined by the World Health Organization: physical, verbal, and sexual [1], as well as common perpetrators for each category. The observed period was the 12 months preceding the completion of the survey, except for the question about robbery since it does not happen as often as other types of violence. The timeframe of 12 months was determined to minimize the possibility of recall biases. In addition, several other studies used the same time frame so that comparisons were possible.

This study included pharmacists, pharmacy technicians, interns, managers, and directors. Countries included in the study were Croatia, Serbia, Bosnia and Herzegovina, and Montenegro, as the roles of community pharmacy teams in their healthcare systems are quite similar. The sample collected was a convenience one due to the limitations of the research, as explained in paragraph 5.4. The structure of the sample encompassed national, geographical, ethnic, cultural, political, and organizational diversity. In all selected countries, there were both small family pharmacies and pharmacy chains from both the private and public sectors.

A survey in an online Google Form was sent to the pharmacy directors and/or owners of 34 pharmacy organizations across the four countries. Altogether, 24 organizations were included in the study, as 7 invited organizations did not provide any feedback to confirm their interest in conducting the research, and 3 institutions declined to participate. The questionnaire was distributed in the Croatian language since the residents of the included countries fully understand the language, and clarity and relevance for all four countries were confirmed by the groups of experts in the adaptation process. Ethical approvals for the research protocol were obtained from each pharmacy organization’s ethics committee coordinator or owner. The study was conducted in accordance with the ethical standards of the Declaration of Helsinki and approved by the Ethics Committees of institutions included in this research (pharmacy organizations and pharmacy chains). An explanation letter with clarification of the purpose of the study along with the survey link was sent to the pharmacy staff after approval was obtained. It was clearly stated that filling out the form implies consent for participating in the research. Data were collected anonymously during 26 days in November and December 2023, with one reminder sent.

The statistical analysis was performed using the licensed program STATISTICA 6.1 StatSoft Inc., Tulsa, OK, USA. Descriptive data is presented in frequency tables, and the interrelationship of descriptive answers is analyzed using cross-tabulation tables. Answers ranked on a five-point Likert scale were analyzed using descriptive statistics (mean, median). The relationship between descriptive and ordinary answers was tested by *t*-test, and the correlation between ordinary answers was analyzed using Spearman’s Correlation Coefficient and regression analysis.

The statistical testing was performed at a statistical significance level of 95% (*p* < 0.05 represents a statistically significant result).

## 3. Results

### 3.1. Sample Description

Altogether, 732 pharmacists and pharmacy technicians working in community pharmacies completed the questionnaire. The structure of the sample according to age, gender, working position, country, and type of pharmacy ownership are shown in Table 1.

### 3.2. Exposure of Staff in Community Pharmacies to Different Types of Workplace Violence in Preceding 12 Months

Table 2 shows that more than 80% of pharmacy staff were exposed to verbal violence in the workplace, while about 20% of pharmacy staff were exposed to physical and sexual violence.

Table 3 shows the mean and median values of answers to the question of how often pharmacy staff were exposed to physical, verbal, and sexual violence at their workplace by each mentioned person perceived as perpetrator. Answers are ranked on a Likert scale: 1 = It did not happen to me, 2 = Several times, 3 = Once a month, 4 = Once a week, 5 = Almost every day.

In the case of physical, verbal, and sexual violence, the most common perpetrator was the patient or client.

A T-test shows that female and male pharmacy staff were equally exposed to physical violence (*p* = 0.748), female staff were statistically significantly more exposed to verbal violence than male (*p* = 0.026), and female staff were more exposed to sexual violence, but this difference is not statistically significant (*p* = 0.096; Table 4).

Furthermore, there is no statistically significant difference between age groups (*p* > 0.05) or between countries in relation to exposure to physical, verbal, and sexual violence (*p* > 0.05).

### 3.3. Pharmacy Staff Communication about Experienced Violence

To determine to whom pharmacy staff report each type of experienced violence, a multiple-choice question was formed where it was possible to choose more than one answer. Altogether, five choices were offered. Out of 146 respondents who experienced physical violence at least once in the previous 12 months, 240 answers were obtained. For verbal violence, 584 respondents gave 940 responses, and for sexual violence, 150 respondents provided 214 responses. More than 60% of respondents mostly shared with their family/friends about all types of violence (Table 5). Reporting violence to superiors and police was rare. Only 19.2% of participants talked to superiors about physical violence, 24.7% about verbal violence, and 8% about sexual violence. Respondents’ experience of reporting to police showed 15.8% for physical violence, 4.5% for verbal violence, and 2.7% for sexual violence.

### 3.4. Pharmacy Staff Experience with Workplace Robberies

The rating of pharmacy staff members’ experiences with workplace robberies was examined on a Likert scale where 1 meant “not traumatic at all” and 5 “extremely traumatic”. A total of 193 pharmacy staff reported experiencing robbery at the workplace during their total working experience in pharmacy; out of that, 39% experienced it once, 30% several times, and 31% reported it happened during the night or when they were not present in the pharmacy. Table 6 shows that 52.4% of pharmacists who experienced one or more robberies during their work experience consider it to be a traumatic or extremely traumatic experience (4 and 5 on the Likert scale). The mean value of answers was 3.6; that is, on average, pharmacists considered robbery a traumatic experience.

Most pharmacy staff reported that they did not receive any kind of support from their employer nor any other help after experiencing a robbery, as follows:

93.3% did not have guaranteed days off from their employer after the robbery.

95.9% did not use sick leave after the robbery.

94.3% did not use nor were offered any other type of help (e.g., psychological counseling, help from a psychotherapist or psychiatrist, etc.). There was no statistically significant difference between private and state pharmacy owners, nor between owner occupations in relation to employee support.

### 3.5. Pharmacy Staff Sense of Safety

Pharmacy staff members’ feeling of safety at workplace was also checked on a five-point Likert scale. There was a statistically significant negative correlation between exposure to physical, verbal, and sexual violence and the feeling of safety in the workplace (*p* < 0.001). Those who were more often exposed to violence in the workplace had a lower sense of safety in the workplace. Pharmacists and pharmacy technicians who worked in a pharmacy where a procedure for dealing with cases of violence was implemented had a statistically significantly higher feeling of safety than respondents who worked in pharmacies that did not have this procedure or instructions (*p* < 0.001). It was also found that 31% of participants did not know whether there was a procedure in their organization or not. There was no difference in the feeling of safety between participants who had received some kind of training on the topic of workplace violence and those who had not received any training. Pharmacy staff of all age groups, all working positions, and in all included countries rated themselves as “very good” when de-escalating violent events (more than 50% answered 4 and 5 on a five-point Likert scale). Those who attended the training in de-escalating found themselves “very good” at defusing violent situations more often than others, which was statistically significant (*p* < 0.001).

Only 3.3% of pharmacy staff worked in pharmacies that employed a security guard, which is inadequate data for any reliable conclusions.

**Figure 1:**
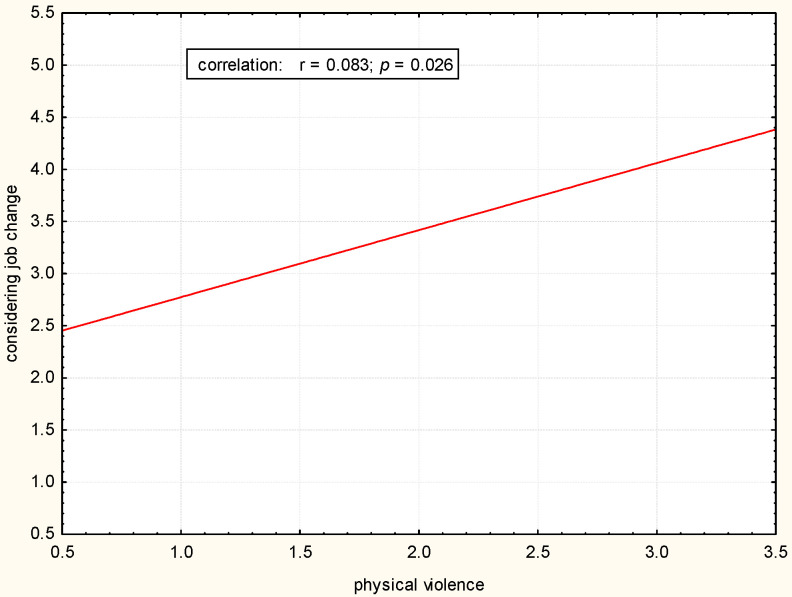
Correlation between considering job change and physical violence.

**Figure 2:**
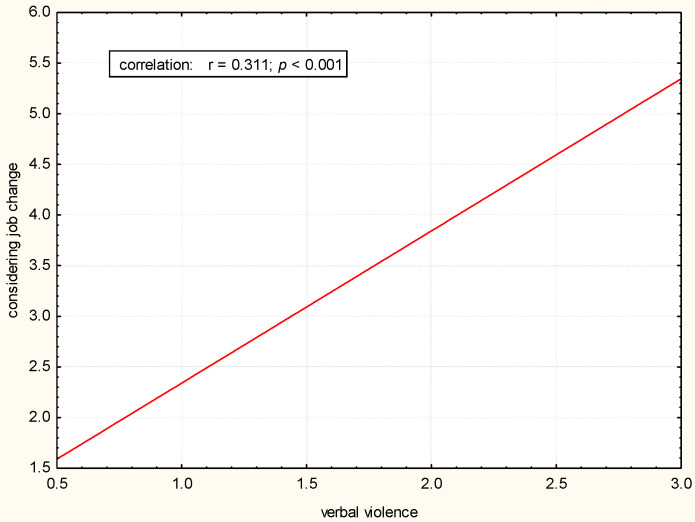
Correlation between considering job change and verbal violence.

**Figure 3:**
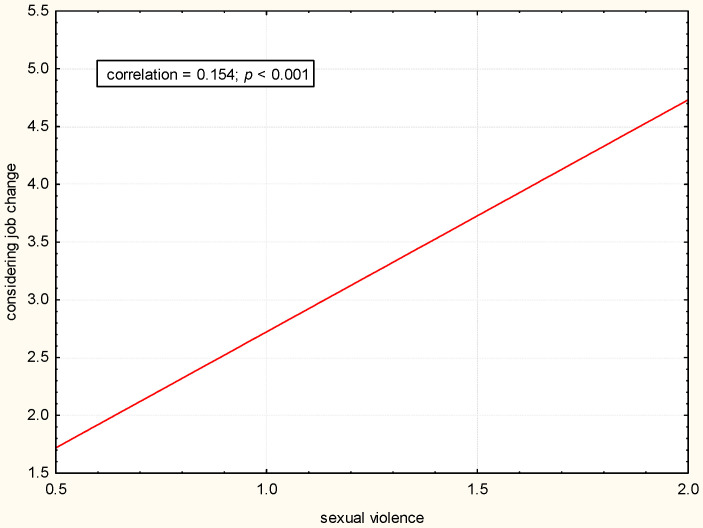
Correlation between considering job change and sexual violence.

There was a statistically significant positive correlation between exposure to physical, verbal, and sexual violence and considering changing the job (*p* < 0.05). The greater the exposure to violence, the more often a job change was considered (Figure 1, Figure 2 and Figure 3).

The intention to leave the community pharmacy in the preceding 12 months was checked on a five-point Likert scale where 1 stood for “completely disagree” and 5 “completely agree” with the statement “Over the past 12 months, I have considered job change and leaving the community pharmacy several times”. In all age groups, there were more than 3% of respondents who thought about changing their job often (4 and 5 on the Likert scale), except for those aged 61 years old and older (4.2%).

On a five-point Likert scale, participants were asked to express their agreement with the statement “After a traumatic event at work (e.g., armed robbery), I would benefit from talking to a professional (psychologist, psychotherapist or psychiatrist)” where 1 stood for “completely disagree” and 5 “completely agree”. For all three types of violence examined, pharmacy staff agreed they would benefit from some form of psychological help after the violent event (Table 7). Results were similar among different age groups, different working positions, and in all countries.

## 4. Discussion

### 4.1. Incidence of External WPV Compared with Other Studies

The first working hypothesis that the pharmacy staff is most exposed to verbal violence was confirmed, with more than 80% of participants confirming the experience of verbal violence in the last 12 months. This aligns with a recent systematic review on WPV toward pharmacists [15] that showed that verbal violence is the most frequent type of violence in community pharmacies. There were no significant differences among age groups, and prevalence in all four countries included in our research was very similar. The conclusions comparing gender differences were not relevant as the proportion of males in the sample was less than 10%. Women were more exposed only to verbal violence (statistically significant), which did not confirm our hypothesis that female pharmacy staff would be more exposed to all types of violence. It is necessary to conduct research on a sample with a greater proportion of male respondents to form valid conclusions. There are studies showing that women are more prone to violence [1], but data is inconsistent [15]. Another hypothesis that was confirmed and aligned with other studies from Ireland, the UK, Australia, Nigeria, and Egypt was that the patients/clients are recognized as the most common perpetrators of violence [16,17,18,19,20,21]. This was confirmed for all three types of violence and in all four included countries from the SEE region in the study.

### 4.2. Ethical Considerations Regarding Reporting Sexual Violence and Managing Consequences of Robberies

Concerning reporting of violent incidents, the study’s findings that the pharmacy staff in most cases talked to someone about verbal violence experienced, and the least about sexual violence, have partly confirmed the study’s hypothesis. The number of sexual violence reports to the police was also low. Sexual violence is not as obvious as physical or verbal, so it is often unnoticed. People report it significantly less because of embarrassment and fear of being seen as unable to cope with it or consider it as part of the job [1,29]. Ethically, this should be considered as a challenge to employers to engage victims of sexual violence to start reporting those incidents.

Another hypothesis that the victims of violence would talk about it mostly with their work colleagues was confirmed, as studies have shown previously in Australia, Nigeria, and Europe [17,20,23]. An especially important finding was that the number of reports to supervisors was low, probably because the previous experience has shown that in more than 90% of cases, there was no structured support for victims of violence, even in traumatic WPV, such as robberies, which confirmed another study hypothesis. Other relevant studies from other countries are aligned with these findings [10,17,22,23].

A total of 26% of participants have experienced a robbery throughout their career; 30% of them more than once. Robberies were analyzed separately due to the complexity, as different forms of violence can be involved. It is a form of violence that is specific to the community pharmacy environment and has the greatest impact on the individual and the team. Given the importance of this experience and the low recall bias, this question captured the respondents’ entire working experience. The percentage of pharmacies employing a security service is very low. It is common to employ a security guard after repeated incidents, but preventative measures are rarely in place. The survey did not include questions about other possible protective measures. There is no standardized equipment or general security protocols, but some pharmacies have a silent alarm for the police, video surveillance, screens, etc. Further analysis is required to describe those details more accurately. The hypothesis that robbery would be considered a very traumatic experience was confirmed by more than half of the participants. Moreover, the experience of robbery was considered traumatic even if the respondent was not present in the pharmacy during the incident. That is, therefore, an extremely important topic to address, since the robberies are specific to community pharmacies in the healthcare sector.

The ethical decision to better support pharmacy staff after they experience traumatic events should be considered, as the majority of participants in all age groups and working positions stated that it would be the preferred response in any type of violent event. Employers are important stakeholders for providing the appropriate risk management approach [29]. There is a need for further research into the type of support that is most useful to offer.

### 4.3. Feeling Safe Working in Community Pharmacy

The study’s hypothesis that there is a negative correlation between feeling safe at the workplace and previous violence exposure for all three types of violence was also confirmed; previous studies with nurses and physicians have already shown the same [24,25]. The literature demonstrates that implemented protocols and support in cases of exposure to violence are important in ensuring safety at workplaces [26,27].

In the study sample, 26.7% of pharmacy staff stated that they have an official protocol established for dealing with violence, but 31% do not even know whether it exists or not. Uninformed staff indicates the gaps in organizational communication culture as well as general unawareness regarding the importance of dealing with workplace violence. Lack of information and a weak perception of this issue are also an ethical concern. It could create insecurity and uncomfortable feelings among work colleagues in community pharmacy settings, as the results of the study indicate that the existence of an official procedure is associated with a higher perceived sense of security [26,27]. Only 15.4% of participants in the study have received training on coping with violent situations, but there is no significant difference in the estimated sense of safety between those who have received education and others (hypothesis partially confirmed). The details of the type and quality of attended education are unknown, as well as the qualifications of the trainers. The literature shows the benefits of different types of training for physicians working in emergency departments in dealing with violent events [30]. It remains to further investigate the effect of training on dealing with violence in the community pharmacy setting. Only 3.3% of respondents work in a pharmacy with a security guard; thus, making conclusions about how this finding relates to a sense of safety was not possible.

The support for violent events should be more integrative than just formal. Providing education and a protocol are important. However, even more important are the communication culture, continuous support, and protective atmosphere, which should be well maintained in pharmacy organizations and companies for their employees to feel safe.

### 4.4. Considering Job Change as a Result of Exposure to Violence in Community Pharmacy

The majority of the study participants (62.4%) consider the everyday job in pharmacy stressful. A total of 35.9% of pharmacy staff in the study have considered changing their job during the last 12 months, and this finding has a positive correlation with exposure to all three types of violence. Most of the participants considered themselves well-skilled in de-escalating violent incidents, especially those who have been educated. Since there is a considerable gap between high rates of violent events and self-perception of the pharmacy staff as being very skilled in handling violent events, it is likely that community pharmacy staff members have overestimated their competence and knowledge on this topic. From individual answers to open comments at the end of the questionnaire, the study has indicated that many participants were not aware that specific situations are considered violent. Therefore, it is important to raise awareness of workplace violence recognition, as well as address and prevent it. Future investigations might take into consideration the content, structure, and impact of educational activities to better understand what the best model could be to address ethical questions regarding exposure to violence in the community pharmacy setting.

### 4.5. Advantages and Limitations of the Study

This is the first study among pharmacy staff in SE Europe describing their experience with workplace violence. Even if it is considered extremely important, it is still a neglected topic. Introducing this type of research will provide new insights into the topic. The collected data might be useful for creating a multi-dimensional approach and defining the starting point to create ethical occupational strategies for the pharmacy setting in SEE.

There is a lack of awareness of the importance of internal and external violence in the workplace. More than 27% of the organizations invited to participate in the study did not respond, or they explicitly decided not to participate. Reasons were not shared with the researchers, but it indicated that in many cases WPV was avoided from being analyzed and discussed as an important problem. Ethically, this could be seen as a concern. The benefit of health workers’ protection and safety should be set as the highest priority. The questionnaire used in the study investigated the frequency of internal violence as well. However, the data were not presented in this paper because they were extensive.

Underreporting of violence is a worldwide problem, but it is especially pronounced in the traditional patriarchal societies in the SEE region. These cultures do not encourage reporting, and the procedures are often complex and time-consuming. Individuals must cope with the system without any organizational support, often facing re-traumatization, such as blaming the victim of violence instead of the assailants. In addition, the awareness of violence in the workplace, as well as connected risk factors, effects, and consequences, is low. Many healthcare workers consider it just an expected part of their job. Robberies, on the other hand, are rightly recognized as acts of violence and are, therefore, regularly reported. However, they are usually reported for insurance purposes. Little or no support was offered to abused staff. There are no statistics on pharmacies in particular; only the total number of robberies in a year is available. There is a need for a systematic approach to WPV in terms of awareness, recognition, appropriate response, support, and prevention. 

The design of the study was cross-sectional, so it was not possible to determine causal relationships between the variables investigated.

Conclusions were made on self-reported data collected through a self-administered survey, so recall bias is possible. The frequency of violence could be either overestimated or underestimated.

There were no data available on the exact number of pharmacy technicians in the countries of the SEE region. Furthermore, the sample was a convenience one; therefore, there is the possibility of sampling bias.

In Serbia and Croatia, there were some campaigns against workplace violence in pharmacies that began during the period when the study was ongoing, so awareness among participants that it would be important to start considering strategies against violence was high [31,32]. 

This study shows the rates of violence in the post-pandemic period when there was a medicine shortage. This study also shows that an increasing number of pharmacists are leaving the healthcare setting, and the workload is growing. In contrast to other studies, this is the only one that includes both pharmacists and pharmacy technicians. It also provides additional data that was not previously available in the studies on pharmacists, such as the data on the relationship between experiences of violence in community pharmacy and the consideration of changing jobs as well as on the relationship between experiences of violence and feelings of safety in the community pharmacy workplace were not found in the available literature.

### 4.6. Possible Solutions to Address Consequences of Violence in Community Pharmacy

The benefits of training and skills for de-escalating violent situations and coping with stress have been well documented in the literature. However, workplace violence is a very complex problem. This study shows that the responsibility for managing workplace violence should be distributed among several stakeholders involved. The findings on the experience with robberies clearly showed that institutional support from the manager or owner of the pharmacy is necessary. It can be assumed that institutional support would be very helpful in the event of any other violent incident as well. Support from the institution was also linked to a percepted sense of safety, which is the basis for the provision of high-quality healthcare. The type of support offered could be diverse (e.g., time off, sick leave, counseling by a psychologist or psychiatrist), but further research is needed to investigate it more precisely.

Regarding prevention strategies, an analysis of risk factors for WPV in community pharmacies could be useful for additional solutions to ensure safe workplaces. It would be helpful to establish protocols and procedures for dealing with violent situations. It is important that all staff are familiar with the protocols. Risk factors were also addressed in the survey conducted for this research. However, a detailed analysis is beyond the scope of this paper and will be the objective of another one. In 2016, the United States Occupational Safety and Health Administration (OSHA) published Guidelines for Preventing Workplace Violence for Healthcare and Social Service Workers [33]. This document contains a number of recommendations for procedures and policies to be implemented. It could be an example of how to help reduce workplace violence in the health and social care sector.

Another important responsibility of pharmacy managers is to encourage their staff to report any kind of violent incident. The reporting rates are currently very low, so there is an opportunity for improvement.

Altogether, a shift of the paradigm in dealing with WPV is needed. An active role by the managers and owners of community pharmacies could have a significant impact on preventing, reporting, and successfully managing violence in the workplace. This could possibly influence the emerging trend of personnel leaving the pharmacy profession. However, further longitudinal studies should be conducted to determine causality since this survey included only participants working in the community pharmacies, excluding those who already left the community pharmacy.

Since the common perpetrators of violence were patients or customers, the public presents an important stakeholder as well. Another action point, therefore, could be taken up in the future through public health and social media campaigns, promoting different types of educational materials to inform the public about the role of community pharmacists and their teams in healthcare in general. A similar conclusion was identified at the summit titled “Implementing Solutions: Building a Sustainable, Healthy Pharmacy Workforce and Workplace”, 2023, in Arlington, Virginia, USA, organized by the American Pharmacists Association (APhA), the American Society of Health-System Pharmacists (ASHP), and the National Association of Boards of Pharmacy (NABP). Participants—including pharmacy professionals from independent and chain community pharmacies, health-system pharmacies, boards of pharmacy (regulators), schools and colleges of pharmacy, and professional pharmacy associations—identified many action items, and one of them was “Educate patients and the public on the value of diverse practice settings (e.g., medication-related roles within the pharmacy workforce) that improve patients’ and public health” [34]. Future research and publications should bring more clarity and describe possible interventions to reduce the consequences of violence in community pharmacies.

## 5. Conclusions

The lack of understanding, recognition, and management of the exposure of pharmacy staff to violence in community pharmacies in SE Europe was obvious. There is a need for a structured approach to addressing violence in pharmacies, including organized support for pharmacy staff. Achieving the quality of patient care, despite dealing with violent individuals or situations daily, is one of the greatest ethical challenges for healthcare providers in community pharmacies to be empowered.

## Figures and Tables

**Table 1:** Sample structure (*n* = 732).

	Count	%
**Age (years)**		
24 or less	44	6.0
25–44	498	68.0
45–60	166	22.7
61 or more	24	3.3
**Gender**		
Female	664	90.7
Male	67	9.2
Other	1	0.1
**Working position**		
Director of the pharmacy	39	5.3
Pharmacy manager	254	34.7
Pharmacist	189	25.8
Pharmacy technician	242	33.1
Intern	8	1.1
**Work experience**		
Less than 1 year	21	2.9
1–5 years	177	24.2
6–14 years	265	36.2
15–24 years	178	24.3
25 years or more	91	12.4
**Country**		
Croatia	296	40.4
Serbia	292	39.9
Bosnia and Herzegovina	77	10.5
Montenegro	67	9.2
**Pharmacy ownership**		
Municipal/county/state/cantonal property	115	15.7
Privately owned—an independent pharmacy	36	4.9
Privately owned—a chain of pharmacies or a healthcare facility	581	79.4

**Table 2:** Frequency of exposure of pharmacy staff in community pharmacies to physical, verbal, and sexual violence in preceding 12 months (*n* = 732).

Type of Violence	Count	%
**Physical violence**		
It did not happen to me	586	80.1
Several times by one or more people	139	19.0
Once a month by one or more people	7	0.9
**Verbal violence**		
It didn’t happen to me	148	20.2
Several times by one or more people	562	76.8
Once a month by one or more people	22	3.0
**Sexual violence**		
It didn’t happen to me	582	79.5
Several times by one or more people	150	20.5

**Table 3:** Exposure of pharmacy staff in community pharmacies to physical, verbal, and sexual violence from different perpetrators in the preceding 12 months.

Perpetrator	Physical Violence		Verbal Violence		Sexual Violence	
	Mean	Median	Mean	Median	Mean	Median
Patient or client	1.2	1.0	2.3	2.0	1.2	1.0
Superior	1.0	1.0	1.2	1.0	1.0	1.0
Work colleague	1.0	1.0	1.3	1.0	1.0	1.0
Medical representative	1.0	1.0	1.1	1.0	1.0	1.0
Other healthcare worker who is not a pharmacist	1.0	1.0	1.2	1.0	1.0	1.0
Deliveryman	1.0	1.0	1.1	1.0	1.0	1.0
Pharmacy staff’s member of family	1.0	1.0	1.2	1.0	1.0	1.0
Third person	1.1	1.0	1.1	1.0	1.0	1.0
Total	1.1	1.0	1.3	1.3	1.0	1.0

**Table 4:** Statistical difference between female and male pharmacy staff in relation to exposure to physical, verbal, and sexual violence.

Type of Violence	Mean Female	Mean Male	*p*-Value
Physical violence	1.06	1.07	0.748
Verbal violence	1.32	1.23	0.026
Sexual violence	1.05	1.02	0.096

**Table 5:** Pharmacy staff communication about experienced violence.

Reporting	Physical Violence		Verbal Violence		Sexual Violence	
	Count	%	Count	%	Count	%
To no one	21	14.4	51	8.7	22	14.7
To work colleagues	94	64.4	401	68.7	94	62.7
To superiors	28	19.2	144	24.7	12	8.0
To family/friends	69	47.3	317	54.3	75	50.0
To the police	23	15.8	26	4.5	4	2.7
Skipped question	5	3.4	21	3.6	7	4.7

**Table 6:** Pharmacy staff experience with workplace robberies (*n* = 193).

Rate of the Traumatic Experience	Count	%
1	6	3.1
2	23	11.9
3	63	32.6
4	43	22.3
5	58	30.1
Mean	3.6	

1 = not traumatic at all, 2 = mostly not traumatic, 3 = undecided, 4 = mostly traumatic, 5 = extremely traumatic.

**Table 7:** Pharmacy staff attitudes toward psychological help after violent incident.

Type of Violence	Count	Mean	St. Dev.
Physical violence	146	3.9	1.24
Verbal violence	584	4.0	1.24
Sexual violence	150	4.3	1.05

## Data Availability

Data is unavailable due to privacy and ethical restrictions.

## Supplementary Materials

The following supporting information can be downloaded at: https://www.mdpi.com/article/10.3390/pharmacy12030088/s1, File S1: Questionnaire.
