# Supplementary material for: Exposure of Pharmacists and Pharmacy Technicians to Violence in Community Pharmacies in Southeast Europe: Frequency and Ethical Considerations
Source: Pharmacy (Basel). 2024 Jun 4;12(3):88. doi: 10.3390/pharmacy12030088 (PMC11207363; doi:10.3390/pharmacy12030088)
Supplement: Supplementary file 1 [file pharmacy-12-00088-s001.zip › pharmacy-2874767-supplementary.pdf]

Dear colleagues,

This is a questionnaire used as part of the study “Exposure to workplace violence against pharmacists and pharmacy technicians in community pharmacies in South and Eastern Europe: Frequency, risk factors and ethical considerations”. The objective of the study is to determine the frequency and types of violent acts to which pharmacists (pharmacists and pharmacy technicians) are exposed in their workplace, with the main focus on the violent acts they experience from people outside the pharmacy team.

This idea came about as a project for my thesis at the School of Personal Development Pharma Expert at the end of last year, the aim of which is to contribute to the profession and the community. The participants of this study are pharmacists from the countries of the region: Croatia, Serbia, Bosnia and Herzegovina and Montenegro.

All data will be collected anonymously and used solely for the purposes of this study, and will not be disclosed to third parties. By completing this questionnaire, you consent to the use of the data collected. The questionnaire should take about 10 minutes to complete. Thank you for taking the time to complete the questionnaire.

For further questions and information on the research results, please contact istrazivanjewpv@gmail.com.

Monika Popčević, M. Pharm.

Group 1: Tick one answer from the choices given:

A1. Age

a. 24 or less
b. 25-44
c. 45-60
d. 61 or more

A2. Gender

a. Female
b. Male
c. Other

A3. Current working position

a. Director of pharmacy
b. Pharmacy manager
c. Pharmacist
d. Pharmacy technician
e. Intern

A4. How many years of professional experience do you have in community pharmacy?

a. Less than 1 year
b. 1-5 years

c. 6-14 years
d. 15-24 years
e. 25 years or more

A5. Which country do you work in?

a. Croatia
b. Serbia
c. Bosnia and Herzegovina
d. Montenegro

A6. How is the ownership of the pharmacy/healthcare facility where you work structured?

a. Municipal/county/state/cantonal ownership
b. Privately owned - an independent pharmacy
c. Privately owned - a chain of pharmacies or a healthcare facility

A7. Is the owner of the pharmacy in which you are employed a pharmacist?

a. Yes
b. No
c. I don't know

A8. Has your pharmacy/facility implemented a formal procedure or procedural guidelines for handling occupational violence against employees? (For example, record forms, contact person in the collective, free psychological support for coping with trauma after a violent event)

a. Yes
b. No
c. I don't know

A9. Have you ever attended a course on how to recognize violence in the workplace, how to de-escalate (defuse) conflict situations or similar?

a. Yes
b. No
c. I don't know

A10. Does the pharmacy you work in currently employ a security guard?

a. Yes
b. No

Group B investigates the frequency and type of violent acts at work that you have experienced in the last 12 months.

Workplace violence means any situation in which workers are subjected to abuse, threats or attacks in connection with their work, including the travel to and from work, which constitute a direct or indirect risk to their safety, well-being or health (European Commission) .

This questionnaire will investigate the frequency of physical, verbal and sexual violence against pharmacists in the workplace itself, excluding travel to work.

B1. Physical violence means the use of physical force against another person or group of people that results in physical, sexual or psychological harm. This includes hitting, slapping, stabbing, shooting, pushing, pinching, throwing objects, etc.

Please specify how often you have been subjected to physical violence by each listed person in your workplace in the last 12 months. Do not include incidents related to sexual harassment as they will be addressed separately.

	It did not happen to me	Several times	Once a month	Once a week	Almost daily
A patient or a customer	1	2	3	4	5
A superior	1	2	3	4	5
A colleague	1	2	3	4	5
Medical representative	1	2	3	4	5
Other health care practitioner that is not a pharmacist (doctor, nurse, dental practitioner, etc.)	1	2	3	4	5
Courier	1	2	3	4	5
A member of your family (e.g. your current or former partner)	1	2	3	4	5
Third party (no service or business-related visit to the pharmacy, e.g. a robber)	1	2	3	4	5

B2. Who did you talk to about the physical violence you experienced? (Multiple answers possible; if you have not experienced physical violence, continue with the next question)

a. To nobody

b. To your colleagues

c. To your superiors

d. To your family/friends

e. To the police

B3. Verbal violence means a form of psychological violence that includes threats, insults, belittling, shouting, intimidation, humiliation, the use of abusive speech or profanities.

Please specify how often you have been subjected to verbal violence by each listed person in your workplace in the last 12 months. Do not include incidents related to sexual harassment as they will be addressed separately.

	It did not happen to me	Several times	Once a month	Once a week	Almost daily
A patient or a customer	1	2	3	4	5
A superior	1	2	3	4	5
A colleague	1	2	3	4	5

Medical representative	1	2	3	4	5
Other health care practitioner that is not a pharmacist (doctor, nurse, dental practitioner, etc.)	1	2	3	4	5
Courier	1	2	3	4	5
A member of your family (e.g. Your current or former partner)	1	2	3	4	5
Third party (no service or business-related visit to the pharmacy, e.g. a robber)	1	2	3	4	5

B4. Who did you talk to about the verbal violence you experienced? (Multiple answers possible; if you have not experienced verbal violence, continue with the next question)

a. To nobody

b. To your colleagues

c. To your superiors

d. To your family/friends

e. To the police

B5. Sexual violence means any sexual act, attempted sexual act, unwanted sexual comment or suggestion directed against a person and their sexuality that can be committed by another person regardless of their relationship to the victim or their situation (WHO). This also includes sexual harassment: Unsolicited sexual comments and verbal innuendos, unsolicited phone calls and touching, undue attention, standing too close, staring, emotional stalking, etc.

Please specify how often you have been subjected to sexual violence by each listed person in your workplace in the last 12 months.

	It did not happen to me	Several times	Once a month	Once a week	Almost daily
A patient or a client	1	2	3	4	5
A superior	1	2	3	4	5
A work colleague	1	2	3	4	5
Medical representative	1	2	3	4	5
Other health care worker who is not a pharmacist (doctor, nurse, dentist, etc.)	1	2	3	4	5
Deliveryman	1	2	3	4	5
A member of your family (e.g. your current or former partner)	1	2	3	4	5
Third party (no service or business-related visit to the pharmacy, e.g. a robber)	1	2	3	4	5

B2. Who did you talk to about the sexual violence you experienced? (Multiple answers possible; if you have not experienced sexual violence, continue with the next question)

a. To nobody

b. To your colleagues

c. To your superiors

d. To your family/friends

e. To the police

B7. During your entire employment in the pharmacy to date, have you ever witnessed a robbery at work?

NO YES once YES several times YES, but outside working hours (during the counter shift or at night)

*For those who experienced it:

*B8. Rate how traumatic the experience was for you according to the scale (if you had several experiences, choose the most traumatic one)

Hardly traumatic at all 1 2 3 4 5 Extremely traumatic

*B9. After the robbery, were you given any days off by your employer?

YES NO

*B10. Did you take sick leave after the robbery?

YES NO

*B11. Did you seek any other support after the robbery (e.g. psychological counseling, psychotherapy, etc.)?

YES NO

C. In the following questions, use your experience to rate on a scale the extent to which each situation or event represents a significant risk factor for the outbreak of violent events in your workplace. All claims refer to violence by external visitors to the pharmacy (violence that occurs internally within the pharmacy team or in relationship with superiors is excluded).

Group 1:

External factors	Insignificant	Less significant	Neither insignificant nor significant	Significant	Very significant
Shortage of medicines	1	2	3	4	5

Crowded pharmacy and long queues of people awaiting service	1	2	3	4	5
Patients uninformed on the functioning of the healthcare system (e.g. lack of understanding of the differences between prescription types, requests to pick up prescription drugs without a prescription or without a health insurance card, ignorance about the difference between the basic and supplementary list of drugs, lack of information about the importance of compulsory insurance, methods of e-prescription, etc.).	1	2	3	4	5
Other healthcare workers uninformed on the functioning of a pharmacy (e.g. incorrectly issued prescriptions or mobility aids, prescribing medications on a repetitive prescription that is only issued on non-repetitive prescription, recommending patients to purchase prescription drugs without a prescription)	1	2	3	4	5
Unjustified patient requests for the improper dispensing of medicines (e.g. requests for the sale of prescription drugs without a correct prescription, claims for the repeated dispensing of drugs that are only registered for one-time dispensing, etc.).	1	2	3	4	5
Attempts to return medicines and medical products	1	2	3	4	5
Unannounced, unplanned or excessively long visits from business partners	1	2	3	4	5

Group 2:

Pharmacy internal factors	Insignificant	Less significant	Neither insignificant nor significant	Significant	Very significant
Workforce deficiencies and therefore increased workload for available pharmacy workers	1	2	3	4	5
Incorrect dispensing of medicines by the pharmacist (e.g. incorrectly dispensed medication, incorrect number of packaging or incorrect packaging of the medication, etc.)	1	2	3	4	5
Communication failures of pharmacists towards patients (e.g. verbal impoliteness, rushing, failure to warn of the expiry date when dispensing medicines or dietary supplements, failure to check whether a person has allergies, etc.)	1	2	3	4	5
Communication failures within the team (e.g. incomplete information on ordered items, returns, discounts, unclear distribution of tasks, etc.)	1	2	3	4	5
Female pharmacist	1	2	3	4	5

Group 3:

Social and political contexts	Insignificant	Less significant	Neither insignificant nor significant	Significant	Very significant
Poor economic situation and inflation (e.g. increased prices for non-prescription medicines, changes to the list of medicines: conversion of	1	2	3	4	5

medicines from the basic list to the supplementary list, increased surcharges, etc.)					
Sensationalist media coverage (e.g. in the event of product recall, shortages of medicines or medical products, etc.)	1	2	3	4	5
Negative evaluation of pharmacists based on conspiracy theories about the pharmaceutical industry as greedy and immoral.	1	2	3	4	5

D. Use the scale to rate whether the following statements reflect your experience.

	I completely disagree	I disagree	Neither agree nor disagree	I agree	I completely agree
I feel safe at my workplace.	1	2	3	4	5
My job is stressful.					
Over the past 12 months, I have considered changing job and leaving the community pharmacy several times.	1	2	3	4	5
I am skilled at defusing conflict situations in the workplace.	1	2	3	4	5
After a traumatic event at work (e.g. armed robbery), I would benefit from talking to a professional (psychologist, psychotherapist or psychiatrist).	1	2	3	4	5

E. Additional comment:

Thank you for taking time to participate in this questionnaire!
